# Assessing Local Risk of Rifampicin-Resistant Tuberculosis in KwaZulu-Natal, South Africa Using Lot Quality Assurance Sampling

**DOI:** 10.1371/journal.pone.0153143

**Published:** 2016-04-06

**Authors:** Christine L. Heidebrecht, Laura J. Podewils, Alexander Pym, Thuli Mthiyane, Ted Cohen

**Affiliations:** 1 Clinical and Biomedical Tuberculosis Research Unit, Medical Research Council, Durban, South Africa; 2 Division of Tuberculosis Elimination, Centers for Disease Control and Prevention, Atlanta, Georgia, United States of America; 3 KwaZulu-Natal Research Institute for Tuberculosis and HIV, Durban, South Africa; 4 School of Laboratory Medicine and Medical Sciences, University of KwaZulu-Natal, Durban, South Africa; 5 Epidemiology of Microbial Diseases, Yale School of Public Health, New Haven, Connecticut, United States of America; Jawaharlal Nehru University, INDIA

## Abstract

**Background:**

KwaZulu-Natal (KZN) has the highest burden of notified multidrug-resistant tuberculosis (MDR TB) and extensively drug-resistant (XDR) TB cases in South Africa. A better understanding of spatial heterogeneity in the risk of drug-resistance may help to prioritize local responses.

**Methods:**

Between July 2012 and June 2013, we conducted a two-way Lot Quality Assurance Sampling (LQAS) study to classify the burden of rifampicin (RIF)-resistant TB among incident TB cases notified within the catchment areas of seven laboratories in two northern and one southern district of KZN. Decision rules for classification of areas as having either a high- or low-risk of RIF resistant TB (based on proportion of RIF resistance among all TB cases) were based on consultation with local policy makers.

**Results:**

We classified five areas as high-risk and two as low-risk. High-risk areas were identified in both Southern and Northern districts, with the greatest proportion of RIF resistance observed in the northernmost area, the Manguzi community situated on the Mozambique border.

**Conclusion:**

Our study revealed heterogeneity in the risk of RIF resistant disease among incident TB cases in KZN. This study demonstrates the potential for LQAS to detect geographic heterogeneity in areas where access to drug susceptibility testing is limited.

## Introduction

In South Africa, the latest published estimates from a nationally representative tuberculosis (TB) drug resistance survey date back to 2002. At that time, 1.8% of new (treatment-naïve) TB cases and 6.7% of previously treated cases (i.e. individuals requiring re-treatment) were infected with multidrug-resistant (MDR) TB, defined by resistance to first-line drugs isoniazid and rifampicin [1]. While these risks are relatively are low compared to Eastern Europe (where the risk among new cases may exceed 25% and the risk among previously treated cases routinely exceeds 50%), South Africa is ranked sixth worldwide for absolute numbers of incident TB cases and therefore even low proportions of drug resistance translate into a high number of cases requiring treatment and risk for transmission of MDR TB [1].

An epidemic of extensively drug resistant (XDR) TB in Tugela Ferry, in the KwaZulu-Natal (KZN) province of South Africa, captured global attention in 2006–7 [2]. Transmission of XDR TB strains was identified in both nosocomial [3] and community settings [4], raising questions about the extent to which highly drug-resistant forms of disease were being transmitted in the province. Subsequent analysis of laboratory data revealed a large burden of detected MDR TB (~2,800 cases in 2007), representing a greater than 10-fold increase in the number of detected MDR TB cases since 2001 [5]; however these statistics underestimated the burden of MDR TB disease because use of culture and drug susceptibility testing (DST) was limited within the province. Nevertheless, given the overall incidence of TB in the province and the high rates among tested patients, it is likely that KZN has among the highest absolute incidence rates of MDR and XDR TB in the world.

Due primarily to variable access to DST, routine surveillance data in South Africa has not historically been used to investigate local heterogeneity in the burden or risk of drug-resistant TB. Population-representative drug resistance surveys, such as the one performed in 2002, can provide overall average estimates of the risk of drug resistant TB disease. However, these studies are resource-intensive (and thus infrequently performed) and do not provide information at a level of geographic granularity that may be valuable for programmatic decision making [6,7].

Lot quality assurance sampling (LQAS) is an alternative survey approach that allows for the classification of an area’s risk of resistance among newly diagnosed TB cases; this approach prioritizes the desire to identify areas that exceed pre-specified thresholds over a precise estimate of the proportion of drug resistance among new TB cases [8,9]. This sampling approach is designed to provide a quick situational assessment to inform programmatic action. LQAS has previously been utilized to estimate coverage or burden of a range of health events including immunization [10,11], malaria control efforts [9], parasitic infections [8], and MDR TB [6].

We applied LQAS in several jurisdictions of KZN to explore its potential to assess spatial variation in the risk of rifampicin (RIF)-resistant TB among new TB cases in KZN. This evaluation was undertaken to gain a better understanding of the prevalence of drug-resistant TB in specific geographic areas and help inform local programs on activities and allocation of resources to prevent ongoing transmission.

## Methods

### Research Sites and Study Population

This study was conducted in three health districts of KZN: Sisonke, uMkhanyakude, and Zululand. Health districts were chosen through stakeholder consultation based on their perceived high (Zululand, uMkhanyakude) and low (Sisonke) drug-resistant TB burden. Seven sites across the three health districts were selected for inclusion; each site included a district hospital and all of the health facilities in the hospital catchment area. In each site, sputum samples are routinely collected from individuals with presumptive TB presenting at community clinics and hospitals and sent to a central district hospital laboratory for testing. Systematic surveillance of drug-resistance has not been conducted in this region. Rollout of the Xpert^®^ MTB/RIF (GeneXpert) assay, facilitating routine collection of data describing RIF resistance, was initiated in South Africa in 2011 [12]; however, widespread implementation had not been achieved at the time that this study was conducted.

Two of the seven sites selected for inclusion in the study were not using GeneXpert at the time of recruitment; LQAS was conducted prospectively in these sites to allow us to collect and process samples with GeneXpert at the time of treatment initiation. The remaining five laboratories had transitioned from smear microscopy to GeneXpert as the primary TB diagnostic tool, and LQAS was thus conducted retrospectively as additional patient samples were not required.

The study population comprised newly-diagnosed, bacteriologically-confirmed patients with TB who were ≥18 years of age and who had never previously received more than one month of treatment for TB. Patients who were unable to provide informed consent, had received more than two doses of TB treatment for current TB disease at the time of GeneXpert diagnostic testing, or were incarcerated at the time of recruitment were ineligible.

### Determining Sample Size and Decision Rule for LQAS

**Fig 1:**
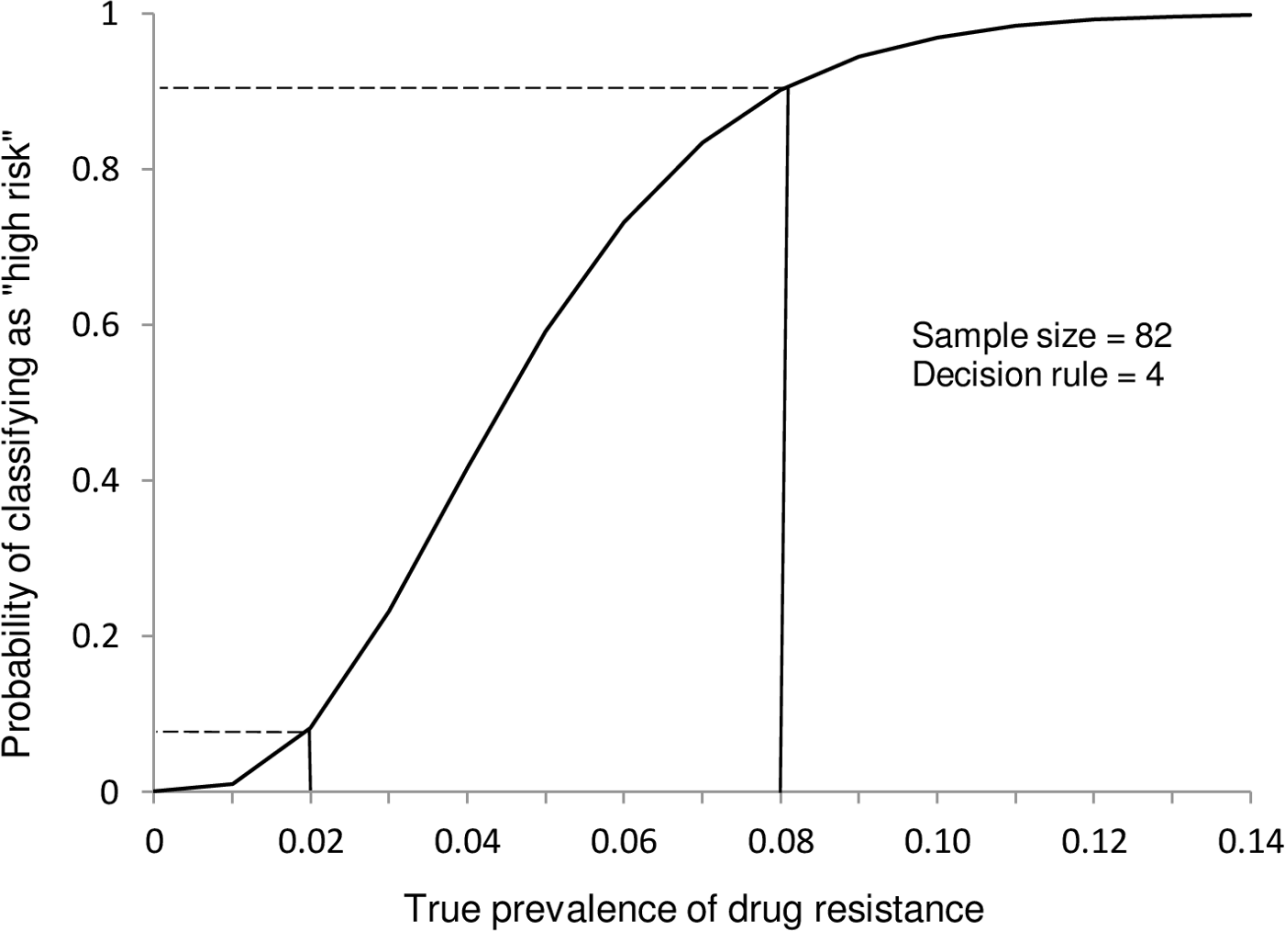
Operating characteristic curve for a sample size of 82 and decision rule of 4. The operating characteristic curve demonstrates that a sample size of 82 new TB cases and a decision cut-off of 4 achieves the desired misclassification properties (<10% misclassifications at a true prevalence of resistance of 8% and at a true prevalence of resistance of 2%).

Stakeholder guidance and previous drug resistance surveys informed our sample size and decision rule calculations. We determined that a sample size of 82 isolates per site and a corresponding decision rule of four drug-resistant cases would allow us to achieve our desired LQAS performance requirements: less than 10% probability of erroneously classifying an area as “high risk” if the true proportion of RIF resistance was 2% or lower, and less than 10% probability of erroneously classifying an area as “low risk” if the true proportion of RIF resistance was greater than 8% (Fig 1). Accordingly, areas in which drug resistance was detected in four or more isolates, among 82, would be classified as “high risk”. RIF resistance was identified using GeneXpert.

### Recruitment and Data Collection: Prospective Sites

In sites where GeneXpert was not yet available, TB diagnosis was determined by sputum smear microscopy. Consecutively-tested patients with smear-positive disease were identified daily at each central hospital laboratory and assessed for eligibility by study personnel. Patients whose laboratory records indicated that they were <18 years of age or incarcerated were excluded immediately; study staff continued eligibility assessment of all remaining patients at the clinic where their sputum sample(s) had been collected. Date of birth and treatment history were reviewed in each patient’s clinic record, and patients who were <18 years of age, classified as retreatment patients, or who had received more than two doses of TB treatment for the current infection were excluded. (Patients who had received more than two doses of treatment prior to collection of a specimen for GeneXpert testing were excluded due to concerns about the impact that longer treatment duration might have on laboratory results). Study staff arranged to meet eligible patients at the clinic–often when the patient returned to receive TB results–or at their home. Eligible patients were asked to provide written informed consent, complete a short questionnaire, and provide a sputum sample. Previous treatment status was based on record review and verbal report. Specimens were transported to a research laboratory in Durban where they underwent GeneXpert testing, (liquid—MGIT) culture, and, when culture positive, drug susceptibility testing (DST). When RIF resistance was detected, study personnel communicated these results to each jurisdiction’s TB coordinator and primary health care supervisor. Recruitment continued until 82 GeneXpert-positive isolates had been collected at each site.

### Recruitment and Data Collection: Retrospective Sites

GeneXpert was used as the primary TB diagnostic tool in the remaining five jurisdictions. This avoided the need for prospective recruitment and collection of a second sputum specimen. Lab and clinic records were reviewed to determine eligibility; patients who appeared to be eligible were contacted telephonically by study staff. Upon giving verbal informed consent, patients were asked to confirm whether or not they had been treated for TB in the past. Patients who had received more than one month of TB treatment at any point in the past or more than 2 doses of TB treatment prior to the date of the diagnostic specimen or who could not be reached were excluded. We recruited all eligible patients consecutively at these sites to mimic the approach used for the prospective enrollment until 82 patients with GeneXpert-positive results had been identified in each site.

### Statistical Analysis

All analyses were performed using Stata version 13 (College Station, TX, USA). We evaluated differences in demographic and clinical characteristics between cases that were eligible and ineligible for inclusion, and between patient cohorts at study sites using Pearson’s chi-square test and Fisher’s exact test (when one or more cells contained values of ≤5). Differences in median age between sites were assessed using the Wilcoxon rank-sum test.

### Ethics Approval

Approval for this study was granted by the South African Medical Research Council’s Ethics Committee, KZN’s Department of Health, the Institutional Review Boards at Brigham and Women’s Hospital, and the U.S. Centers for Disease Control and Prevention. Formal support was also obtained from each health district in which the study was conducted.

## Results

### Prospective Sites

Between July 2012 and March 2013, 513 patients with positive sputum smears were assessed for eligibility at two sites in Zululand Health District: Vryheid Hospital and Nkonjeni Hospital. Twenty-three weeks of recruitment were required to reach our desired sample size in Vryheid and 22 weeks were required in Nkonjeni.

**Fig 2:**
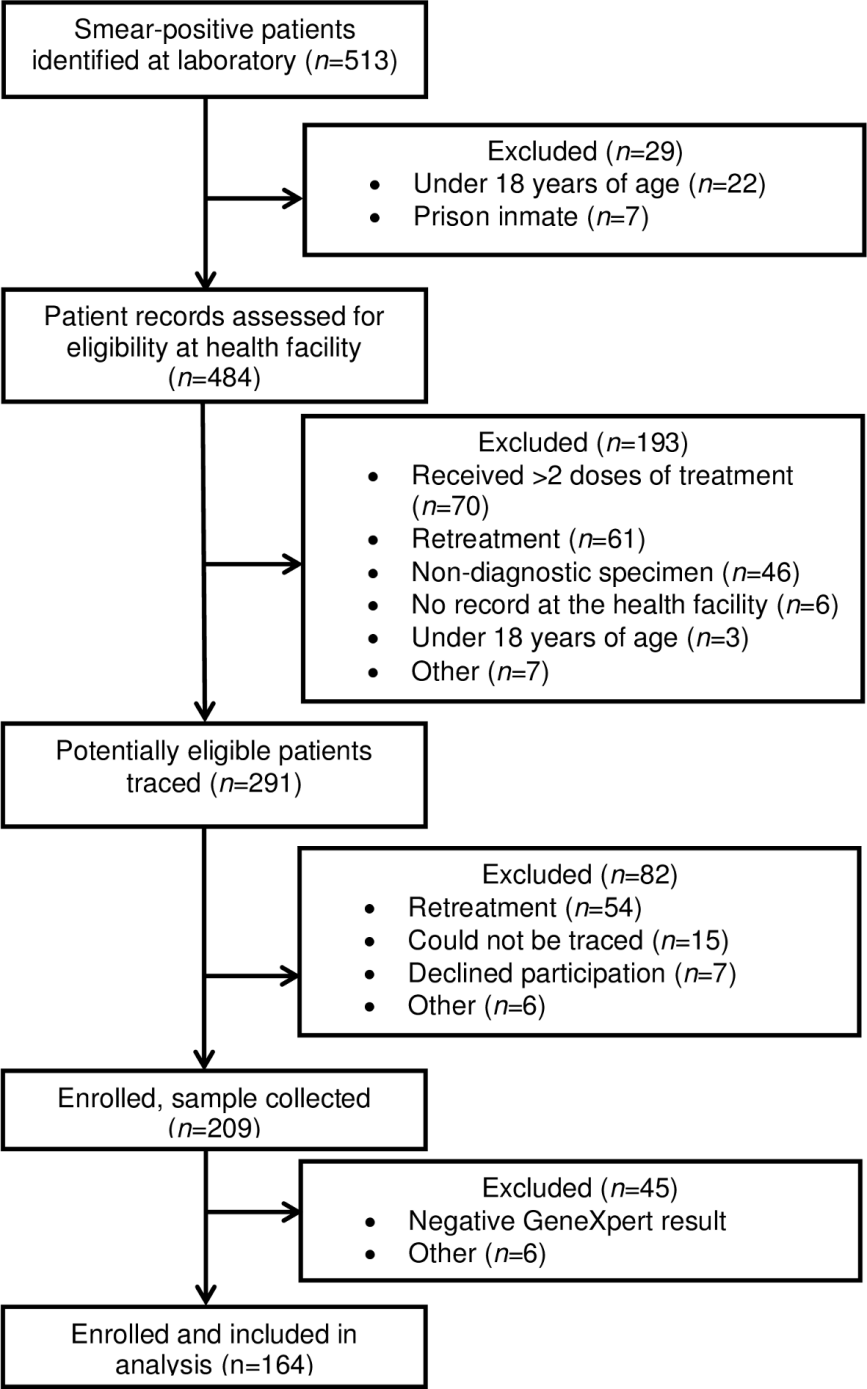
Eligibility and enrollment of TB cases included at sites that enrolled prospectively.

Among patients assessed for eligibility, a total of 349 were deemed ineligible or were excluded from the analysis because their sputum sample did not yield a positive GeneXpert result; 164 patients were included in the analytic sample (Fig 2). Fifty-four (18.5%; 54/291) patients with confirmed TB status whose clinic records did not indicate that they had received TB treatment in the past informed research staff that they had previously received treatment for TB.

Eligible and ineligible patients did not differ with respect to sex in either site, but enrolled participants in Nkonjeni had a younger median age (29 years; IQR: 26–43) than ineligible patients (36 years; IQR: 28–46) (*p* = 0.01), and smear grades assigned to the diagnostic sputum samples of eligible patients tended to be higher than those assigned to ineligible patients (*p* = 0.04). There were no significant differences in age or smear grade between eligible and ineligible patients in Vryheid (data not shown).

Characteristics of enrolled patients with positive GeneXpert results did not differ significantly between study sites, with the exception of smear grade (Table 1). A higher proportion of participants in Nkonjeni had low grades of smear positivity compared with participants in Vryheid.

**Table 1:** Participant characteristics at prospective sites, KwaZulu-Natal Province, South Africa (n = 164).

		TOTAL (n = 164) n (%)	Vryheid (n = 82) n (%)	Nkonjeni (n = 82) n (%)	*p*-value*
Sex					
	Female	68 (41.5)	31 (37.8)	37 (45.1)	0.34
	Male	96 (58.5)	51 (62.2)	45 (54.9)	
Age (years)					
	18–24	29 (17.7)	12 (14.6)	17 (20.7)	0.55
	25–34	63 (38.4)	31 (37.8)	32 (39.0)	
	35–44	45 (27.4)	27 (32.9)	18 (22.0)	
	45–54	20 (12.2)	9 (11.0)	11 (13.4)	
	55+	7 (4.3)	3 (3.7)	4 (4.9)	
Smear grade					
	Scanty	18 (11.0)	10 (12.2)	8 (9.8)	<0.01
	1+	60 (36.6)	18 (22.0)	42 (51.2)	
	2+	36 (22.0)	12 (14.6)	24 (29.3)	
	3+	50 (30.5)	42 (51.2)	8 (9.8)	
HIV status**					
	Negative	47 (32.6)	25 (32.5)	22 (32.8)	0.38
	Positive	82 (56.9)	37 (48.1)	45 (67.2)	
	Don’t know	5 (3.5)	5 (6.5)	0	
	Don’t want to disclose	10 (6.9)	10 (13.0)	0	
Ever served time in prison					
	No	132 (80.5)	66 (80.5)	66 (80.5)	1.00
	Yes	32 (19.5)	16 (19.5)	16 (19.5)	
Been admitted to hospital in the last year					
	No	135 (82.3)	64 (78.0)	71 (86.6)	0.15
	Yes	29 (17.7)	18 (22.0)	11 (13.4)	
Ever worked in a mine					
	No	147 (89.6)	74 (90.2)	73 (89.0)	0.80
	Yes	17 (10.4)	8 (9.8)	9 (11.0)	
Lived in a household with someone with TB in the last year					
	No	126 (76.8)	65 (79.3)	61 (74.4)	0.23
	Yes	35 (21.3)	14 (17.1)	21 (25.6)	
	Don’t know	3 (1.8)	3 (3.7)	0	

*Pearson’s chi-square (excludes unknown values); Fisher’s exact test used when one or more cells contained values ≤5

**Self-reported; among patients who had been tested for HIV (*n* = 144; 77 in Vryheid, 67 in Nkonjeni)

**Fig 3:**
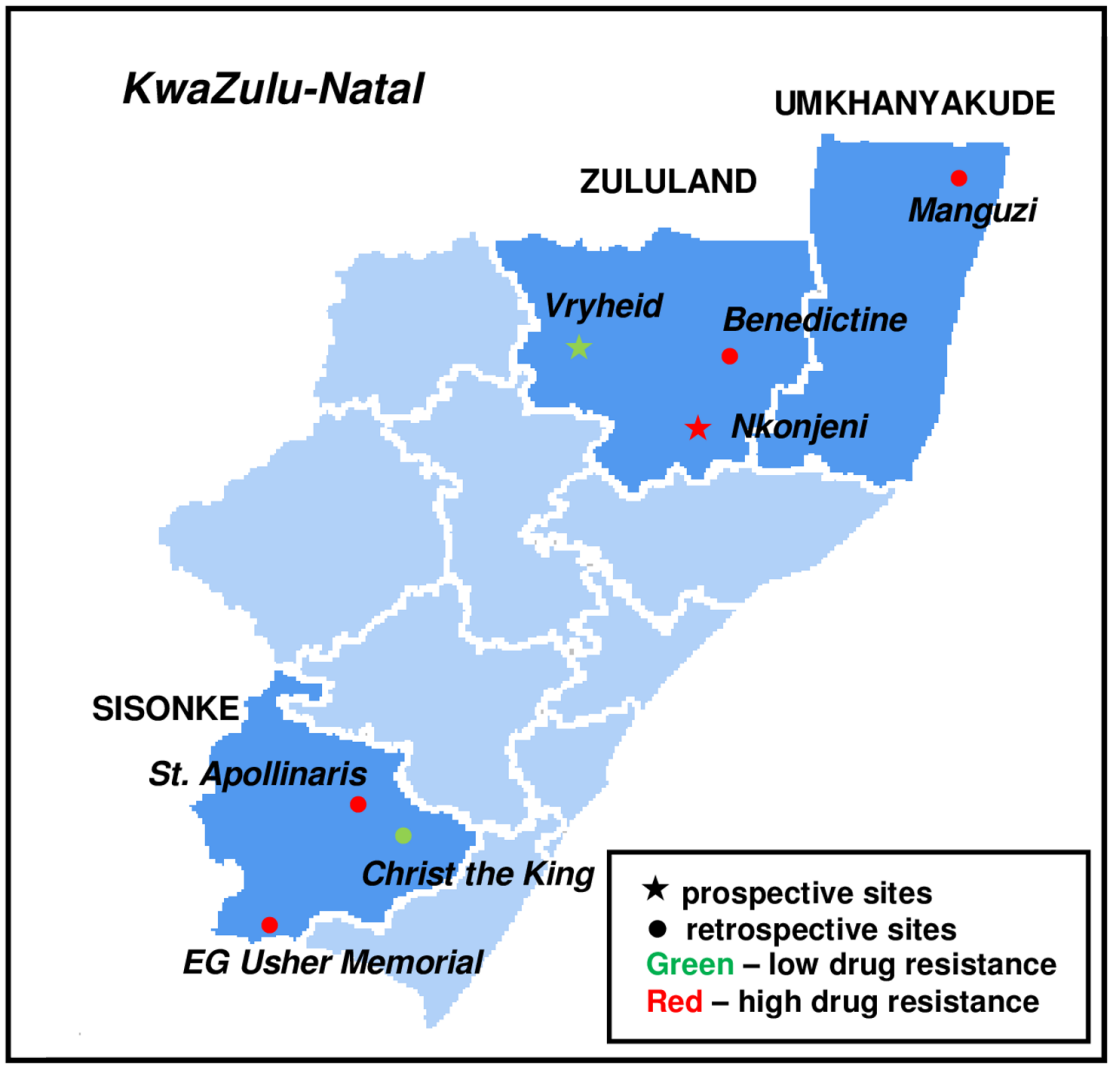
Study sites and risk classifications. Classifications of areas in KwaZulu-Natal Province, South Africa, as high or low risk for rifampicin resistant TB based on Lot Quality Assurance Sampling and GeneXpert testing. Areas in dark blue were included in the study. *Modified from "Map of South Africa with district borders (2011)" (author*: *Htonl) under a CC Attribution-Share Alike 3*.*0 Unported license*, *with permission from Wikimedia Commons*, *original copyright 2011*. *Available*: https://commons.wikimedia.org/wiki/File:Map_of_South_Africa_with_district_borders_(2011).svg

GeneXpert testing identified RIF resistance among three (3.7%) of the 82 isolates collected in Vryheid, and in Nkonjeni, RIF resistance was detected in five (6.1%) of 82 isolates (Table 2). The former was thus classified as a low-risk area for drug resistance and the latter classified as high risk (Fig 3).

**Table 2:** LQAS classifications: prospective sites (n = 164).

		Vryheid	Nkonjeni
		(*n* = 82) *n*(%)	(*n* = 82) *n*(%)
RIF resistance detected			
	Yes	3 (3.7)	5 (6.1)
	No	79 (96.3)	77 (93.9)
LQAS classification: risk of drug resistance		Low	High

GeneXpert and culture results were available for 162 isolates across both sites; we found 93% agreement between methods. GeneXpert positive/culture negative results comprised the majority of discordant pairs (Table 3). MTB was isolated by culture in isolates from eight patients with resistance based on GeneXpert RIF but RIF resistance was not confirmed by DST in three of these cases (Table 4).

**Table 3:** Line listing of samples with discordance between GeneXpert and MGIT liquid culture for detection of TB.

GeneXpert result	Culture result	Smear result	HIV status	Started Rx prior to sample collection?	Sputum quality
MTB not detected	Positive	Not detected	Negative	No	2ml; mucoid
Positive	MTB not isolated	2+	Negative	No	2.5ml; mucoid
Positive	MTB not isolated	2+	Negative	Yes; received 1 dose	2.5ml; mucoid
Positive	MTB not isolated	2+	Positive (not on ART)	Yes; received 1 dose	1.5ml; semi-mucoid
Positive	MTB not isolated	Not detected	Negative	No	3ml; saliva
Positive	MTB not isolated	1+	Negative	No	<5ml; not viscous/watery
Positive	MTB not isolated	3+	Positive (not on ART)	No	2ml; mucoid
Positive	MTB not isolated	Not detected	Positive (not on ART)	Yes; received 1 dose	2ml; mucoid
Positive	MTB not isolated	1+	Positive (not on ART)	No	2ml; semi-mucoid
Positive	MTB not isolated	Not detected	Has not been tested	No	<5ml; saliva
Positive	MTB not isolated	Not detected	Positive (no response re ART)	No	<5ml; mucoid

**Table 4:** MGIT liquid culture drug-susceptibility testing (DST) results for patients identified as RIF-R by GeneXpert. (Bold font denotes MDR).

	RIF resistant	RIF susceptible	No growth for DST	Other DST results*
Case 1	X			Susceptible: EMB, OFL, KAN, CAP
				Resistant: INH, SM, ETH
Case 2	X			Susceptible to all but RIF
Case 3	X			Susceptible: OFL, KAN, CAP, NIAC
				Resistant: INH, EMB, ETH
				N/A: SM
Case 4	X			Susceptible: SM, OFL, KAN, ETH, CAP
				Resistant: INH, EMB
Case 5	X			Susceptible to all but RIF
Case 6		X		Susceptible to all
Case 7		X		Susceptible to all
Case 8			X	N/A

*INH = isoniazid; EMB = ethambutol; SM = streptomycin; OFL = ofloxacin; KAN = kanamycin; ETH = ethionamide; CAP = capreomycin

### Retrospective Sites

The laboratory records of 847 patients with positive GeneXpert results were retrospectively reviewed in order to achieve our desired sample size in five sites across three health districts: Benedictine Hospital in Zululand; Manguzi Hospital in uMkhanyakude; and St. Apollinaris, EG Usher Memorial, and Christ the King Hospitals in Sisonke. The period over which test results were reviewed varied by site and ranged from four to seven months.

**Fig 4:**
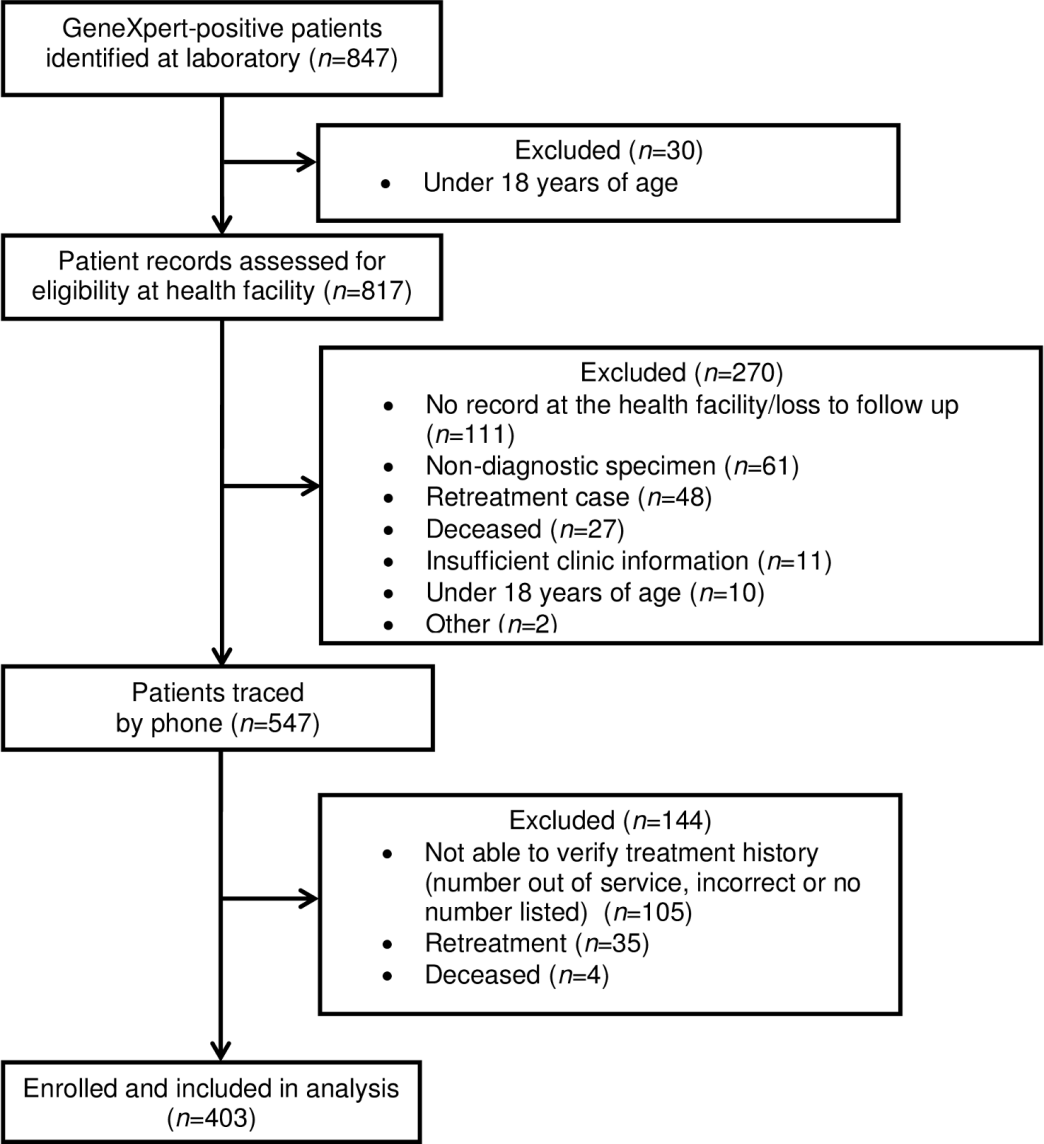
Eligibility and enrollment of TB cases included at sites that enrolled retrospectively.

Of 817 patients who met eligibility criteria based on laboratory records, an additional 270 were excluded: 119 were not eligible (non-diagnostic specimen, retreatment case or <18 years of age); 122 had missing or inadequate health records; 27 had died prior to the time of the assessment; and 2 were excluded for other reasons (Fig 4). An additional 35 patients whose treatment status was documented as “new” in clinic records informed research staff that they had been previously treated for TB. Research staff were unable to verify the eligibility of 109 patients (not able to reach or deceased).

We observed no significant differences between eligible and ineligible patients with respect to sex, age or GeneXpert classification in any of the retrospective sites. There was no significant difference in the proportion of RIF-resistant cases among enrolled patients and those who we could not trace, with the exception of one site, Benedictine. Characteristics of enrolled patients did not differ significantly between sites (Table 5).

**Table 5:** Participant characteristics at retrospective sites, KwaZulu-Natal Province, South Africa (n = 403).

		TOTAL (*n* = 403)	Benedictine (*n* = 82)	Christ the King (*n* = 82)	St. Apollinaris (*n* = 75)	EG Usher Memorial (*n* = 82)	Manguzi (*n* = 82)	*p*-value*
		*n* (%)	*n* (%)	*n* (%)	*n* (%)	*n* (%)	*n* (%)	
Sex								
	Female	206 (51.1)	45 (54.9)	39 (47.6)	37 (49.3)	40 (48.8)	45 (54.9)	0.81
	Male	197 (48.9)	37 (45.1)	43 (52.4)	38 (50.7)	42 (51.2)	37 (45.1)	
Age (years)								
	18–24	79 (19.6)	23 (28.0)	10 (12.2)	14 (18.7)	12 (14.6)	20 (24.4)	0.09
	25–34	140 (34.7)	28 (34.1)	34 (41.5)	22 (29.3)	29 (35.4)	27 (32.9)	
	35–44	84 (20.8)	14 (17.1)	20 (24.4)	12 (16.0)	17 (20.7)	21 (25.6)	
	45–54	54 (13.4)	12 (14.6)	9 (11.0)	11 (14.7)	13 (15.9)	9 (11.0)	
	55+	46 (11.4)	5 (6.1)	9 (11.0)	16 (21.3)	11 (13.4)	5 (6.1)	
GeneXpert classification**								
	Very low	90 (22.3)	23 (28.0)	13 (15.9)	18 (24.0)	21 (25.6)	15 (18.3)	0.50
	Low	107 (26.6)	18 (22.0)	23 (28.1)	24 (32.0)	21 (25.6)	21 (25.6)	
	Medium	136 (33.7)	27 (32.9)	30 (36.6)	24 (32.0)	30 (36.6)	25 (30.5)	
	High	69 (17.1)	14 (17.1)	16 (19.5)	9 (12.0)	10 (12.2)	20 (24.4)	
HIV status***								
	Negative	128 (31.8)	30 (36.6)	23 (28.0)	24 (32.0)	23 (28.0)	28 (34.1)	0.48
	Positive	246 (61.0)	47 (57.3)	56 (68.3)	40 (53.3)	57 (69.5)	46 (56.1)	
	Not recorded/ unknown	29 (7.2)	5 (6.1)	3 (3.7)	11 (14.7)	2 (2.4)	8 (9.8)	

Due to logistical constraints, only 75 eligible individuals were recruited from St. Apollinaris Hospital.

*Pearson’s chi-square (excludes unknown values)

**Based on cycle threshold (Ct) values; the inverse concentration of genetic material in the sample. One sample in Manguzi was not able to be classified.

Very low: Ct >28 Medium: Ct16-22

Low: Ct 22–28 High: Ct <16

***Abstracted from clinic records

Four or more cases of RIF resistance were detected in four sites in the retrospective component of this study; these were thus classified as high-risk areas for RIF resistance (Table 6).

**Table 6:** LQAS classifications: retrospective sites (n = 403).

		Benedictine	Christ the King	St. Apollinaris	EG Usher Memorial	Manguzi
		(*n* = 82)	(*n* = 82)	(*n* = 75)	(*n* = 82)	(*n* = 82)
		*n* (%)	*n* (%)	*n* (%)	*n* (%)	*n* (%)
RIF resistance detected						
	Yes	5 (6.1)	3 (3.7)	4 (5.3)	5 (6.1)	11 (13.4)
	No	74 (90.2)	79 (96.3)	71 (94.7)	75 (91.5)	71 (86.6)
	Indeterminate	3 (3.7)	0	0	1 (1.2)	0
	Unknown	0	0	0	1 (1.2)	0
LQAS classification: risk of drug resistance		High	Low	High	High	High

## Discussion

Lot quality assurance sampling allowed us to detect heterogeneity in risk of RIF-resistant TB across geographic regions of KZN. While the prospective and retrospective components used slightly different inclusion criteria and recruitment approaches, limiting direct comparison of results, we do note that variability in RIF resistance risk among new cases was observed across sites using both study designs. One of two sites in the prospective component was classified as a high-burden area, as were four of five sites in the retrospective component.

In the Manguzi site, located in Umkhanyakude, the northernmost district of KZN, we found a substantially higher risk of RIF resistance than in other sites surveyed. In this site we found that 13.4% new patients (i.e. not previously treated) had RIF-resistant TB, but the small sample size associated with the LQAS survey means that the 95% confidence interval for this sample spans from 6.9% to 22.7%. While the LQAS approach aims to classify the level of risk based on a pre-determined threshold, since our sample was drawn from a consecutive sample of eligible patients, this estimate should be unbiased for the population from which this sample was drawn. We note that our finding of higher risk of resistance than previously reported estimates supports earlier observations of high rates of MDR TB case notifications in this district dating back to 2007. The high risk was noted despite the fact that clinicians in this district may not have been requesting DST for a large fraction of patients with tuberculosis [4].

Striving to rapidly evaluate risk of drug resistance through LQAS–prospectively as well as retrospectively–we based our analyses on GeneXpert results which consequently limited our assessment to rifampicin resistance. LQAS could be applied to assess distribution of risk of MDR TB in the study region, but due to inconsistent DST practices in South Africa this type of evaluation would need to be conducted prospectively with laboratory procedures performed independently of routine health services. Given the strong association of isoniazid resistance with rifampicin resistance, we do not believe that an LQAS evaluation of the risk MDR TB would yield a different assessment than what we have reported based on GeneXpert results. Classifications of other resistance patterns (e.g. XDR) could also be examined through LQAS, but samples sizes and decision rules would need to re-calculated based on stakeholder guidance.

Owing to a number of factors, LQAS was not as rapid an exercise as anticipated when applied prospectively in our study population. A relatively large proportion of patients with smear-positive TB assessed for eligibility were retreatment patients and not eligible, resulting in fewer eligible patients than anticipated. Further, because a second sputum sample was required to perform GeneXpert, culture, and drug susceptibility testing, many patients who would have met inclusion criteria initially were deemed ineligible by the time of recruitment because study staff had been unable to reach them before they had received two doses of treatment. In Nkonjeni, eligible patients were significantly younger and had higher sputum grades than patients who were ineligible; the higher sputum grade is likely a reflection of the high number of patients who were ineligible due to already having initiated anti-TB treatment. Additionally, 45 of the initial 209 (21.5%) enrolled patients were excluded from analysis because their second sample did not yield a positive GeneXpert result. The impact of some of these issues may have been mitigated had LQAS been conducted as a programmatic exercise rather than a research initiative, reducing the time required to reach the desired sample size. Enrollment (including confirmation of treatment history) and secondary sample collection could have been conducted by program staff at the first clinical interaction with every person suspected of having TB; upon availability of microscopy results, GeneXpert testing could be performed on samples produced by patients with positive sputum smear results. Accounting for the proportion of retreatment patients expected among those with unknown status, and assuming that a sample collected on the same day and under the same conditions as a smear-positive sample would have produced a GeneXpert-positive result, we estimate that 92 additional patients would have been eligible for inclusion in Vryheid, and 44 in Nkonjeni, resulting in data collection windows that were reduced by twelve and eight weeks respectively.

In contrast, retrospective LQAS was a rapid exercise but was also subject to the quality of existing clinical records. In order to be confident that we were excluding patients being retreated for TB, we had to obtain additional confirmation of treatment history directly from patients through telephone interviews. A sizable proportion of patients who we were unable to contact had to be excluded from the analysis.

Study limitations included poor patient data availability at two clinics in Benedictine’s jurisdiction; they were insufficient for study purposes and these clinics were excluded from the study. Unmeasured drug resistance among patients who could not be traced in the prospective component of the study may have impacted whether or not the decision rule was reached and thus each area’s assigned LQAS classification. However, these individuals comprised a small proportion of all excluded patients. At Benedictine, a site included in the retrospective component of the study, patients that were not able to be traced were more likely to be RIF resistant than enrolled patients. Since this site was classified as a high-risk area, however, if the untraced patients were able to be contacted it would have increased the proportion estimate of RIF resistance, but would not have altered the study classification of this site. Though overall test agreement was high, we did observe some discordance between GeneXpert and culture results, particularly with respect to detection of RIF resistance. Of the three GeneXpert-identified RIF-resistant cases that were not confirmed by culture, one showed growth failure for DST, which we do not consider a truly discrepancy, and the remaining two demonstrated RIF susceptibility. It is not clear why these results were discordant. Resource limitations precluded gene sequencing and genotyping that may have identified *rpoB* mutations or the presence of multiple strains respectively, suggesting RIF resistance. Conversely, the GeneXpert assay is not 100% specific and one or both of these results may have been false (RIF resistant) positives. Even if this were the case, however, the classification of each site as high or low risk would not differ from those based on GeneXpert results alone. Further, due to regional differences in the timing of implementation of GeneXpert as a primary diagnostic test for TB, the retrospective component included persons who were both smear positive and negative, but we required an initial bacteriologic confirmation of smear positivity at our prospective sites to determine eligibility to obtain and test a second sputum sample with GeneXpert. It is possible that this discrepancy may interfere with our ability to compare the two approaches. However, the LQAS decision rules classifying sites as high or low risk within a local geographic area were based on a proportion, and the definitions of the numerator and denominator were consistent across all sites within the local area. Lastly, the clinical data collected were not originally intended for research use. While we were able to counter known discrepancies with respect to treatment history with patient verification, there may have been other data subject to poor record-keeping that could have impacted study observations.

Several considerations should be made when planning to apply LQAS in a programmatic context. While we continued sampling until the full sample size had been achieved to allow a comprehensive assessment of LQAS, allowing for early stopping when (and if) the decision rule has been reached [3,5] can facilitate prompt public health action in areas deemed high risk. LQAS assessments can also be designed to yield trichotomous classifications, that is, to distinguish between low-, moderate- and high-risk areas [6,13,14], which may further assist program managers in priority-setting activities. The number of sites included in our analysis was sufficient to demonstrate geographic heterogeneity but is likely too few to be of value in public health decision making to prioritize resources and direct programmatic action; a programmatic evaluation should include a more comprehensive set of sub-districts.

Over the past five years, improved access to drug susceptibility testing (including both culture-based approaches and more rapid diagnostic tools such as line probe assays (e.g. INNO-LiPA, Hain) and cartridge based nucleic acid amplification tests (e.g. GeneXpert) have increased the detection of MDR cases throughout South Africa. GeneXpert is now used as a routine first-line diagnostic tool, which means that highly detailed spatial mapping of RIF-resistant TB will be possible in the near future. While universal access to DST may transform South Africa’s response to MDR TB if access to effective treatment can be scaled up to meet the burden which is being revealed by GeneXpert, universal DST is not yet the reality in most high burden TB settings. In such settings, new approaches to investigate the spatial distribution of drug resistance, such as LQAS, may help to prioritize the use of limited resources for MDR TB prevention and control.
